# Polymorphism of the Tryptophan Hydroxylase 2 (TPH2) Gene Is Associated with Chimpanzee Neuroticism

**DOI:** 10.1371/journal.pone.0022144

**Published:** 2011-07-13

**Authors:** Kyung-Won Hong, Alexander Weiss, Naruki Morimura, Toshifumi Udono, Ikuo Hayasaka, Tatyana Humle, Yuichi Murayama, Shin'ichi Ito, Miho Inoue-Murayama

**Affiliations:** 1 The United Graduate School of Agricultural Science, Gifu University, Gifu, Japan; 2 Scottish Primate Research Group, Department of Psychology, School of Philosophy, Psychology and Language Sciences, The University of Edinburgh, Edinburgh, United Kingdom; 3 Wildlife Research Center of Kyoto University, Kyoto, Japan; 4 The Chimpanzee Sanctuary Uto, Sanwa Kagaku Kenkyusho co., Ltd., Uto, Kumamoto, Japan; 5 School of Anthropology and Conservation, University of Kent. Canterbury, Kent, United Kingdom; 6 National Institute of Animal Health, Tsukuba, Japan; 7 Faculty of Applied Biological Sciences, Gifu University, Gifu, Japan; Texas A&M University, United States of America

## Abstract

In the brain, serotonin production is controlled by tryptophan hydroxylase 2 (*TPH2*), a genotype. Previous studies found that mutations on the *TPH2* locus in humans were associated with depression and studies of mice and studies of rhesus macaques have shown that the *TPH2* locus was involved with aggressive behavior. We previously reported a functional single nucleotide polymorphism (SNP) in the form of an amino acid substitution, Q468R, in the chimpanzee *TPH2* gene coding region. In the present study we tested whether this SNP was associated with neuroticism in captive and wild-born chimpanzees living in Japan and Guinea, respectively. Even after correcting for multiple tests (Bonferroni *p* = 0.05/6 = 0.008), Q468R was significantly related to higher neuroticism (β = 0.372, *p* = 0.005). This study is the first to identify a genotype linked to a personality trait in chimpanzees. In light of the prior studies on humans, mice, and rhesus macaques, these findings suggest that the relationship between neuroticism and *TPH2* has deep phylogenetic roots.

## Introduction

Serotonin (5-HT) production is mediated by the rate-limiting enzyme tryptophan hydroxylase (*TPH*) [1], and *TPH2* is preferentially located in the dorsal raphe region of the brain [2] and in the peripheral myenteric neurons of the small intestine [3]. A loss of function mutation (R441H) in human *TPH2* was identified in patients with major depression [4] and a loss-of-function mutation (P447R) in mice was associated with significantly reduced aggressive behavior [5]. Also, psychiatric disorders such as bipolar disorder [6], attention deficit/hyperactivity disorder [7], and suicidality [8], have been associated with *TPH2* gene polymorphisms. Chen et al. (2010) also reported a functional polymorphism in the 3′ untranslated region of the *TPH2* gene in rhesus macaques, which was related to differential hypothalamus-pituitary-adrenal axis functioning, and, among peer-reared infants, was associated with the aggressive behavior [9].

The role of the serotonergic system in neuroticism, depression, anxiety-related traits, and disorders has been extensively studied [10], [11], [12], [13], [14], [15]. The serotonin transporter gene *5-HTT* is the primary target of the most widely used class of psychiatric drugs, the selective serotonin reuptake inhibitors (SSRIs) [12]. A functional polymorphism referred to as the 5-hydroxytryptamine-linked polymorphic region (*5-HTTLPR*) is present in the regulatory region of *5-HTT* gene [12]. The short allele (*S*) of this gene is transcribed less efficiently than the long allele (*L*), resulting in decreased *5-HTT* expression [12]. Lesch et al. found that *5-HTTLPR* was significantly associated with anxiety-related traits: individuals with the SS or *SL* genotype presented higher neuroticism scores than those with the *LL* genotype. Several studies [16], [17], [18], [19] have replicated these results, although studies of the general population failed to demonstrate an association [20], [21], [22], [23], [24], [25], [26].

Previous studies indicated that chimpanzee personality can be defined by five personality domains analogous to those of humans (neuroticism, extraversion, openness, agreeableness, and conscientiousness) and a broad, chimpanzee-specific domain labeled dominance [27], [28], [29]. These personality dimensions are reliable across raters [27], [28], [29]. Moreover, subsequent studies have validated these dimensions by demonstrating their relationship with subjective well-being [30], observed behaviors [31], and neuroanatomy [32].

In a prior study, we found that the *5-HTTLPR* gene in chimpanzees was monomorphic [33]. However, in another study we found a gain-of-function single nucleotide polymorphism (SNP) in chimpanzees at the 1,404^th^ position of the tryptophan hydroxylase 2 (*TPH2*) gene - the adenine (A) nucleotide was substituted with guanine (G) [34]. This substitution results in the replacement of the 468^th^ glutamine (CAG, ch468Q) with arginine (CGG, ch468R). An enzyme activity assay of these genotypes indicated that the capacity of L-5-hydroxytrytophan (serotonin) biosynthesis was significantly higher for ch468R than ch468Q [34].

In the present study we examined the association between the Q468R polymorphism and neuroticism in chimpanzees. Like the short version of the human *5-HTTLPR* gene which is related to human neuroticism [12], ch468R might be related to less efficient serotonin recovery at the synapse. Thus, we predicted that the ch468R allele would be associated with higher levels of neuroticism in chimpanzees.

## Methods

### Samples

The sample consisted of 57 chimpanzees. Of these chimpanzees, 21 were cared for at the Chimpanzee Conservation Center, Guinea, West Africa. These individuals were wild-born orphans, rescued from the illegal pet trade, and in the process of being rehabilitated. Of the 36 remaining chimpanzees, 26 lived in Chimpanzee Sanctuary Uto [35], 5 lived in 3 Japanese zoos, and 5 lived in the Kyoto University Primate Research Institute (see Table 1 for details about the age and sex ratio). For the Guinea sample, DNA was obtained non-invasively via fecal samples. For the Chimpanzee Sanctuary Uto sample, DNA was obtained via blood samples. To minimize suffering, the blood samples were not collected for the purpose of the present study, but as part of routine health examinations. During these examinations chimpanzees were sedated with oral midazolam (1 mg/kg) or droperidol (0.2 mg/kg), and their blood was collected while they were anesthetized with ketamine hydrochloride (7 mg/kg) or a combination of ketamine hydrochloride (3.5 mg/kg) and medetomidine hydrochloride (0.035 mg/kg).

**Table 1:** Summary table of the subjects' sex, age, genotype, and personality *T*-score†.

Samples	*n*	Sex	Age	Genotype	Chimpanzee Personality Trait (mean ± SD *T*-score)					
		M/F	(mean ± *SD* years)	*AA*/*AG*/*GG*	Dom	Ext	Con	Agr	Neu	Opn
Chimpanzee Sanctuary Uto	26	14/12	29.7±3.5	20/4/2	55.5±9.6	47.5±7.3	50.2±9.5	52.0±8.4	43.2±7.4	48.7±4.4
Other Japanese*	10	2/8	36.7±7.5	3/4/3	55.7±9.4	43.2±9.2	50.2±11.5	48.7±11.6	49.8±8.4	44.4±8.4
Guinea	21	11/10	5.4±1.6	12/5/4	50.3±10.4	60.1±5.1	55.8±9.4	58.2±8.0	47.1±8.7	55.1±5.9
Combined	57	27/30		35/13/9						

Note.

† *Mean* = 50 and *SD* = 10. Dom = Dominance, Ext = Extraversion, Con = Conscientiousness, Agr = Agreeableness, Neu = Neuroticism, Opn = Openness.

*Other Japanese chimpanzees include 5 subjects from the Kyoto University Primate Research Institute, 2 subjects from the Higashiyama Zoo, 1 chimpanzee from the Itouzu-no-mori Zoo, 1 chimpanzee from the Kouchi Zoo, and 1 chimpanzee from the Tama Zoo.

This study was carried out within the ethical guidelines and framework of Kyoto University and was approved by the Primate Research Institute, Kyoto University and Chimpanzee Sanctuary Uto (permission numbers P1988-08, P1990-15, P2000-04, P2005-01 and P2006-05). All procedures were conducted according to the second edition of the Guide for the Care and Use of Laboratory Primates (Primate Research Institute, Kyoto University) and the Guideline for Care of Chimpanzees (Chimpanzee Sanctuary Uto). The details of animal welfare and steps taken to ameliorate suffering were in accordance with the recommendations of the Weatherall report, “The use of non-human primates in research”.

### Genotyping

Genotyping of Q468R by PCR-RFLP is described in detail by Hong and his colleagues [34]. Briefly, a pair of PCR primers (TPH2F: 5-TTCTGTTTATTCTGCA-GGGACT-3′ and TPH2R: 5′-TTAGCCAAGCCATGACACAG-3′) were used to amplify the 1404^th^ SNP containing fragments of *TPH2*, which was then digested using a restriction enzyme, HpyCH4V (New England BioLabs, Beverly, MA). The digested fragments were subsequently separated by electrophoresis on a 2.0% agarose gel (see Table 1 for details about genotype frequencies).

### Personality ratings

Chimpanzees were scored by raters using a Japanese translation of the Hominoid Personality Questionnaire [36]. Raters were comprised of researchers or keepers from each chimpanzee facility, i.e. zoos, research institutes, Chimpanzee Sanctuary Uto, or the Chimpanzee Conservation Center. Each rater had a minimum of 2 years of experience with the chimpanzees they rated. As in our prior study [27], raters had no previous practice in rating chimpanzees. The translated version of the questionnaire yielded the same dimensions as the original English-language version and had similar psychometric properties, including high inter-rater reliabilities [36]. For the present study, we defined the six chimpanzee personality domains via unit-weighting and using the definitions described in our previous study [36]. The raw scores were standardized and then converted into *T*-scores (*mean* = 50; *SD* = 10).

### Statistical analysis

To cross-validate results, we first separately tested for the association between the *TPH2* genotype and personality in the chimpanzees living at the Chimpanzee Conservation Center in Guinea and those living in Chimpanzee Sanctuary Uto in Japan. We then tested for this association in all 57 subjects (the combined study sample), which included the 10 chimpanzees living in the Kyoto University Primate Research Institute and Japanese zoos. In our first model we examined the possibility of additive genetic effects. We therefore coded *AA*, *AG*, and *GG* as 0, 1, and 2, respectively. In our second model we examined the possibility of genetic dominance by coding We then tested the dominance genetic mode by comparing individuals with the *AA* genotype (coded 0) and individuals with either the *AG* or *GG* genotype (coded as 1). Chimpanzee personality traits and *TPH2* genotypes were analyzed using linear regressions controlling for sex and age. SPSS (15.0) was used to conduct all analyses.

## Results

Table 2 shows the means of each personality domain score for each genotype for the Chimpanzee Sanctuary Uto, Guinea, and combined samples. Among the chimpanzees living in Chimpanzee Sanctuary Uto, the Q468R allele was significantly related to higher dominance scores in the additive and dominance model; there was also a statistically non-significant trend indicating that this allele was associated with higher neuroticism (see Table 3). These associations were not statistically significant in the Guinea sample; however, the association between the ch468R genotype and neuroticism was similar with respect to direction and effect size (see Table 3). In addition, neuroticism, but not dominance, was significantly associated with ch468R in the total sample (see Table 3) and this association was significant even after a Bonferroni correction for multiple tests (*p* = 0.05/6 = 0.008).

**Table 2:** Mean personality domain scores by genotype for chimpanzees living in Chimpanzee Sanctuary Uto, the sanctuary in Guinea, and the combined sample.

	Genotype					
	*AA*		*AG*		*GG*	
	Mean	*SD*	Mean	*SD*	Mean	*SD*
Chimpanzee Sanctuary Uto						
Dominance	53.05	7.81	61.14	12.66	68.84	9.15
Extraversion	46.59	6.89	45.48	2.36	60.60	6.66
Conscientiousness	50.31	9.70	45.99	5.78	57.14	14.41
Agreeableness	52.34	6.49	42.97	4.14	66.40	13.37
Neuroticism	41.77	7.16	48.54	2.33	46.63	12.13
Openness	48.37	4.32	48.27	4.01	53.08	5.44
Guinea						
Dominance	51.58	11.55	50.33	10.29	46.45	8.23
Extraversion	60.39	5.69	58.96	5.68	60.48	3.04
Conscientiousness	55.89	7.27	56.72	12.30	54.45	13.91
Agreeableness	57.50	7.42	56.62	11.01	62.29	5.91
Neuroticism	45.20	8.26	49.68	11.46	49.49	6.74
Openness	56.53	6.98	52.12	3.47	54.28	4.11
Combined†						
Dominance	52.29	8.84	55.61	12.14	55.93	11.64
Extraversion	51.61	9.38	48.22	10.53	54.97	9.37
Conscientiousness	52.35	9.93	49.52	9.83	55.81	10.72
Agreeableness	54.10	7.51	47.02	11.36	61.74	6.99
Neuroticism	43.34	8.06	51.62	8.64	46.84	7.97
Openness	51.16	6.76	48.20	6.92	49.95	7.83

† The combined samples includes 26 subjects from Chimpanzee Sanctuary Uto, 21 subjects from Guinea, and a total of 10 subjects from the Kyoto University Primate Research Institute (*n* = 5), Higashiyama Zoo (*n* = 2), Itouzu-no-mori Zoo (*n* = 1), Kouchi Zoo (*n* = 1), and Tama Zoo (*n* = 1).

**Table 3:** Effect of Tryptophan hydroxylase 2 polymorphism on chimpanzee personality trait: Linear regression analysis with sex and age as the covariates.

				Additive mode (*AA*, *AG*, *GG*)				Dominant mode (*AA*, *AG*+*GG*)			
				Unstandardized		Standardized		Unstandardized		Standardized	
Sample	*n*	MAF†	HPQ factors	*β*	SE	*β*	*p*	*β*	SE	*β*	*p*
Chimpanzee Sanctuary Uto	26	0.154	**Dominance**	**7.377**	**2.923**	**0.472**	**0.019**	**4.959**	**2.023**	**0.442**	**0.023**
			Extraversion	4.271	2.476	0.360	0.098	1.639	1.780	0.192	0.367
			Conscientiousness	−0.161	3.103	−0.010	0.959	−0.996	2.124	−0.090	0.644
			Agreeableness	1.675	3.041	0.123	0.587	−1.193	2.091	−0.122	0.574
			Neuroticism	4.442	2.501	0.378	0.090	3.304	1.699	0.391	0.065
			Openness	1.013	1.476	0.144	0.500	0.425	1.022	0.084	0.681
Guinea	21	0.310	Dominance	−1.628	2.102	−0.126	0.449	−0.652	1.696	−0.063	0.705
			Extraversion	−0.301	1.488	−0.047	0.842	−0.537	1.179	−0.107	0.655
			Conscientiousness	−0.633	2.275	−0.054	0.784	−0.374	1.813	−0.040	0.839
			Agreeableness	1.975	2.141	0.199	0.369	0.809	1.736	0.102	0.647
			Neuroticism	2.500	2.126	0.231	0.256	2.332	1.668	0.272	0.180
			Openness	−1.391	1.596	−0.188	0.396	−1.514	1.246	−0.258	0.241
Combined*	57	0.272	Dominance	2.171	1.706	0.163	0.209	1.783	1.309	0.174	0.179
			Extraversion	1.027	1.266	0.080	0.421	−0.198	0.979	−0.020	0.841
			Conscientiousness	0.472	1.579	0.036	0.766	−0.391	1.215	−0.038	0.749
			Agreeableness	1.765	1.591	0.141	0.272	−0.589	1.235	−0.061	0.636
			**Neuroticism**	**3.214**	**1.502**	**0.279**	**0.037**	**3.309**	**1.115**	**0.372**	**0.005**
			Openness	−1.008	1.093	−0.110	0.360	−1.124	0.833	−0.159	0.183

Note.

† MAF: minor allele frequency.

*The combined samples includes 26 subjects from Chimpanzee Sanctuary Uto, 21 subjects from Guinea, and a total of 10 subjects from the Kyoto University Primate Research Institute (*n* = 5), Higashiyama Zoo (*n* = 2), Itouzu-no-mori Zoo (*n* = 1), Kouchi Zoo (*n* = 1), and Tama Zoo (*n* = 1). Boldfaced values indicate statistically significant effects (*p*<.05). Underlined values indicate trends (*p*<0.1).

## Discussion

The ch468R allele was associated with higher dominance in a sample of chimpanzees living in Chimpanzee Sanctuary Uto. The association between this allele and dominance was not present among the wild-born sanctuary chimpanzees in Guinea or in the total sample. There was also a statistically non-significant trend suggesting that neuroticism in chimpanzees was associated with the ch468R allele in Chimpanzee Sanctuary Uto. Moreover, while this relationship was not statistically significant, the effect size and direction of the effect were comparable in the chimpanzee sample from Guinea. Finally, in the total sample, there was a significant association between the presence of the ch468R allele and higher levels of neuroticism.

One possible reason for the failure to cross-validate the dominance findings is that the wild-born sanctuary chimpanzees were younger than those at Chimpanzee Sanctuary Uto. As such, the dominance dimension may not yet have as clearly been expressed as in the more mature individuals. A second possible explanation for our failure to cross-validate the dominance findings in the wild sample is because they were separated from their mothers early in life and may have been subjected to other trauma.

There does appear to be evidence of an association between neuroticism and ch468R. This is consistent with earlier studies of humans [4] and mice [5] which found that Q468R mutations are associated with major depression and aggressive behavior, respectively. This is also consistent with a recent study which found that the 3′-untranslated-region polymorphism of *TPH2* in rhesus macaques was associated with aggressive behavior [9]. The chimpanzee *TPH2* polymorphism (Q468R) is a gain-of-function mutation, which increases serotonin biosynthesis [34]. In other words, like the *S* allele of *5-HTTLPR* which has been related to human neuroticism, the ch468R allele of the *TPH2* gene works to increase serotonin storage in the synapse by increasing production of and decreasing the re-absorption of serotonin.

One shortcoming of the present study was the small sample size and thus these results require replications in larger independent samples. A second shortcoming is our poor knowledge of the background of the chimpanzees which prevented us from testing for any gene by environment interaction effects. A third shortcoming is that, while we included a model for dominance effects, the mean neuroticism across genotypes were only suggestive with respect to whether the *G* allele was dominant, though this may reflect the small sample size of each group.

Chimpanzees have highly-developed brains and exhibit a variety of psychological and behavioral traits in their elaborate social interactions. The present study is the first to identify a genotype related to a personality trait in chimpanzees. Understanding differences in the genes responsible for behavioral variation could lead to a better understanding of the evolutionary history of humans and chimpanzee [37], including hominization [33]. The finding of similar associations between the *TPH2* gene and phenotypes related to neuroticism in humans, mice and rhesus macaques suggests that the relationship between neuroticism and *TPH2* has deep phylogenetic origins.

This is the first report of a relationship between a personality trait and genotype in great apes. Genetic markers for behavior may be useful for primate conservation, welfare and management in zoos. Therefore, the association between Q468R polymorphism and neuroticism identified in this study should be a focus of future studies which seek to understand individual differences in chimpanzee personality.
